# Supplementary material for: Lattice-mismatch-free growth of organic heterostructure nanowires from cocrystals to alloys
Source: Nat Commun. 2022 Jun 3;13:3099. doi: 10.1038/s41467-022-30870-y (PMC9166754; doi:10.1038/s41467-022-30870-y)
Supplement: Supplementary file 1 — Supplementary Information [file 41467_2022_30870_MOESM1_ESM.pdf]

Supplementary Information

Lattice-Mismatch-Free Growth of Organic Heterostructure Nanowires from Cocrystals to Alloys

Qiang Lv,[1,2] Xue-Dong Wang,[1]* Yue Yu,[1] Ming-Peng Zhuo,[1] Min Zheng,[2,3]* Liang-Sheng Liao[1,4]*

[1]Institute of Functional Nano & Soft Materials (FUNSOM), Jiangsu Key Laboratory for Carbon-Based Functional Materials & Devices, Soochow University, 199 Ren'ai Road, Suzhou, Jiangsu 215123, PR China;

[2]National Engineering Laboratory for Modern Silk, College of Textile and Clothing Engineering, Research Center of Cooperative Innovation for Functional Organic/Polymer Material Micro/Nanofabrication, Soochow University, Suzhou, Jiangsu 215123, PR China;

[3]Jiangsu Naton Science&technology Co., Ltd, Suzhou Industrial Park, Suzhou, Jiangsu 215123, PR China;

[4]Macao Institute of Materials Science and Engineering, Macau University of Science and Technology, Taipa 999078, Macau SAR, China.

*E-mails:

wangxuedong@suda.edu.cn (X.-D. Wang)

zhengmin@suda.edu.cn (M. Zheng)

lsliao@suda.edu.cn (L.-S. Liao)

Characterizations

(1) The morphology and size of the organic micro/nanostructures were examined by field emission scanning electron microscopy (FESEM, Carl Zeiss, Supra 55, Germany) dropping on glass. One drop of the solution was dropped on a carbon-coated copper grid, and then evaporated. Transmission electron microscope (TEM, FEI company, Tecnai G2 F20, United States) measurement was performed at room temperature at an accelerating voltage of 100 kV. The X-ray diffraction (XRD) patterns were measured by a D/max 2400 X-ray diffractometer with Cu $K\alpha$ radiation (λ = 1.54050 Å) operated in the 2θ range from 5° to 35°, by using the samples on the quartz. Fluorescence images were recorded using a fluorescence optical microscope (Leica, DM4000M, Germany) with a spot-enhanced charge couple device (Diagnostic Instrument, Inc.). The excitation source is a mercury lamp equipped with a band-pass filter (330-380 nm for UV-light). The samples were prepared by placing a drop of solution onto a cleaned quartz, and then evaporated at room temperature. Micro-area photoluminescence (μ-PL) spectra were collected on a homemade optical microscopy. Optical absorption spectra of crystals were determined used a Lambda 950 UV-vis-NIR spectrophotometer. The time-resolved fluorescence decay was measured by a HORIBA JOBTN YVON FLUOROMAX-4 spectrofluorometer. The growth morphologies of BGP-OFN and BGP-TFPA were simulated by using the Materials Studio software, based on the attachment energy theory.

(2) Micro-area photoluminescence spectra measurement. Micro-area photo luminescence (μ-PL) spectra were collected on a homemade optical microscopy[4]. To measure the PL spectra of individual microwire was excited locally with a 375nm laser focused down to the diffraction limit. The excitation laser was filtered with a 375nm notch filter. The light was subsequently coupled to a grating spectrometer (Princeton Instrument, ARC-SP-2356) and recorded by a thermal-electrically cooled CCD (Princeton Instruments, PIX-256E). PL microscopy images were taken with an inverted microscope (Olympus, BX43).

(3) Characterization of the growth process of blue-olive-blue triblock

heterostructure. We carry out a series experiments to investigates growth mechanism of heterostructures by controlling crystal growth in a vacuum oven at room temperature. The preparation of mixed solution following the similar experiment procedure of blue-olive-blue triblock nanowires. Then, in the first stage, the mixed solution is dropped on the glass substrate at room temperature in air, wait for the solvent to evaporate naturally for 3 seconds, and immediately vacuumize with a vacuum pump for 20 seconds to achieve instantaneous stop of crystal growth and obtain a crystal sample of the first stage. In the second stage, the growth process of heterostructures following the similar experiment procedure, in which the evaporation time was set to 10 seconds. In the third stage, the growth process of heterostructures following the similar experiment procedure, and the evaporation time was set to 40 seconds. In the fourth stage, the growth process of heterostructures following the similar experiment procedure, the evaporation time was set to 80 seconds. Finally, the obtained samples of different growth stages were characterized by fluorescence microscopy.

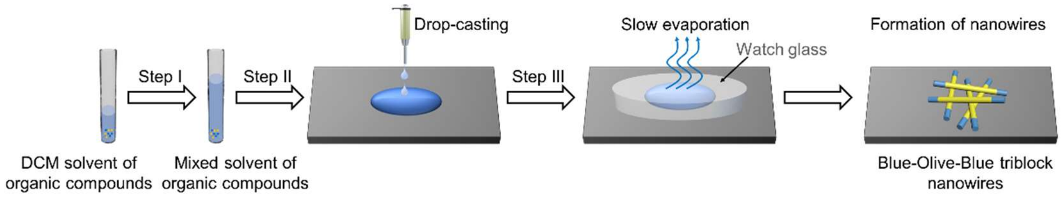

Supplementary Figure 1. Schematic diagram of the preparation procedure. Scheme illustration of the preparation procedure of the blue-olive-blue triblock heterostructures.

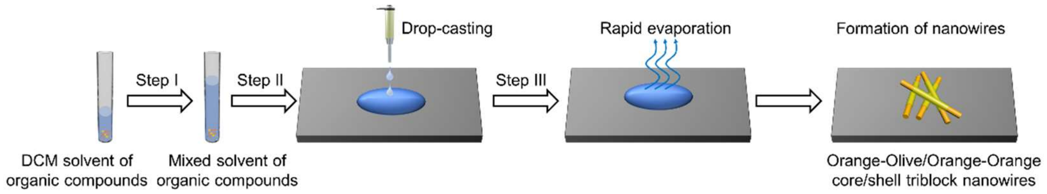

Supplementary Figure 2. Schematic diagram of the preparation procedure. Scheme illustration of the preparation procedure of the orange-olive/orange-orange core/shell triblock heterostructures.

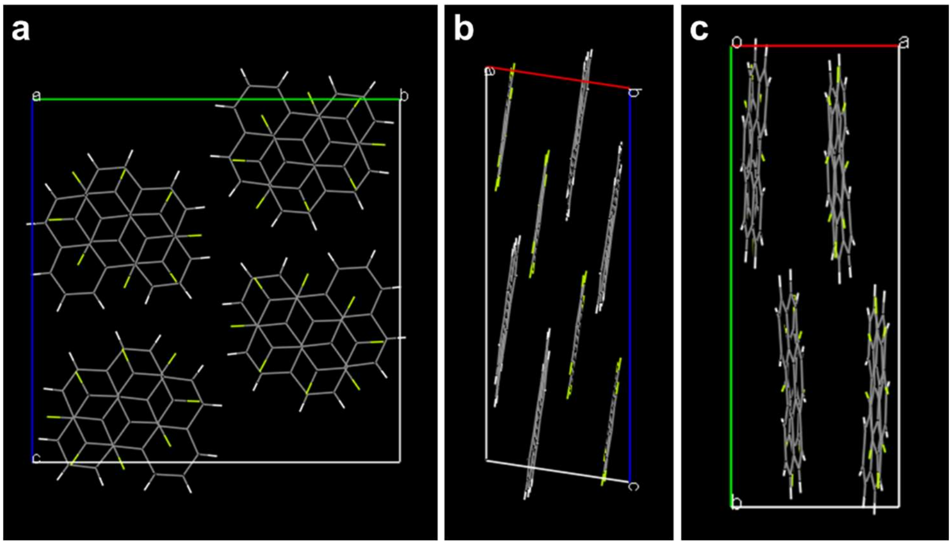

Supplementary Figure 3. Unit cell structure of BGP-OFN cocrystals. Singe crystal packing of BGP-OFN view along a direction (a), view along b direction (b) and view along c direction (c).

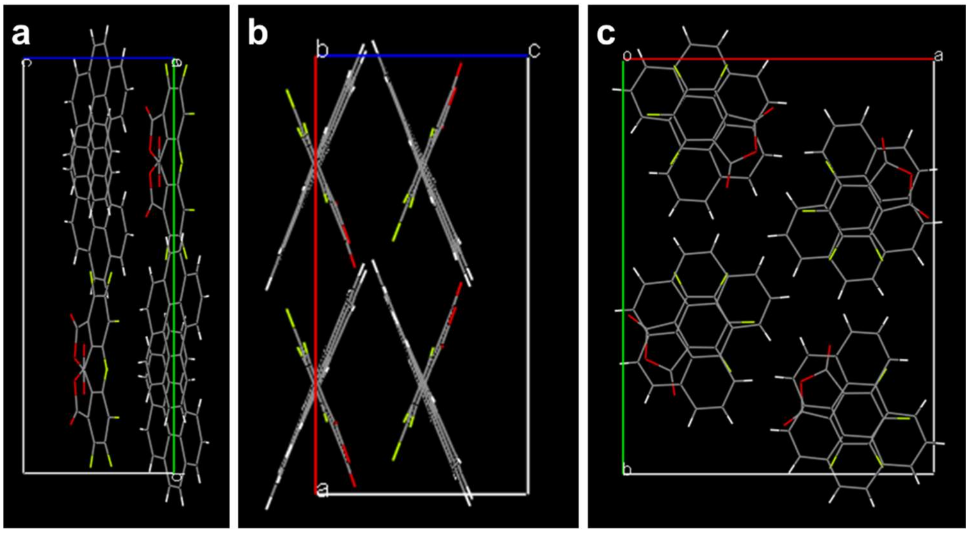

Supplementary Figure 4. Unit cell structure of BGP-TFPA cocrystals. Singe crystal packing of BGP-TFPA view along a direction (a), view along b direction (b) and view along c direction (c).

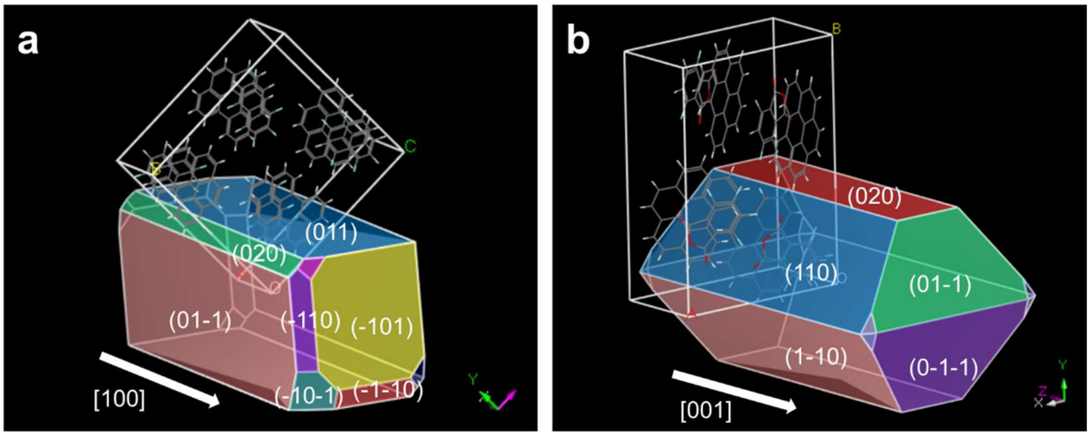

Supplementary Figure 5. Predicted growth morphology of cocrystals. (a) The growth morphology of BGP-OFN cocrystal, crystal growth along [100] direction. (b) The growth morphology of BGP-TFPA cocrystal, crystal growth along [001] direction.

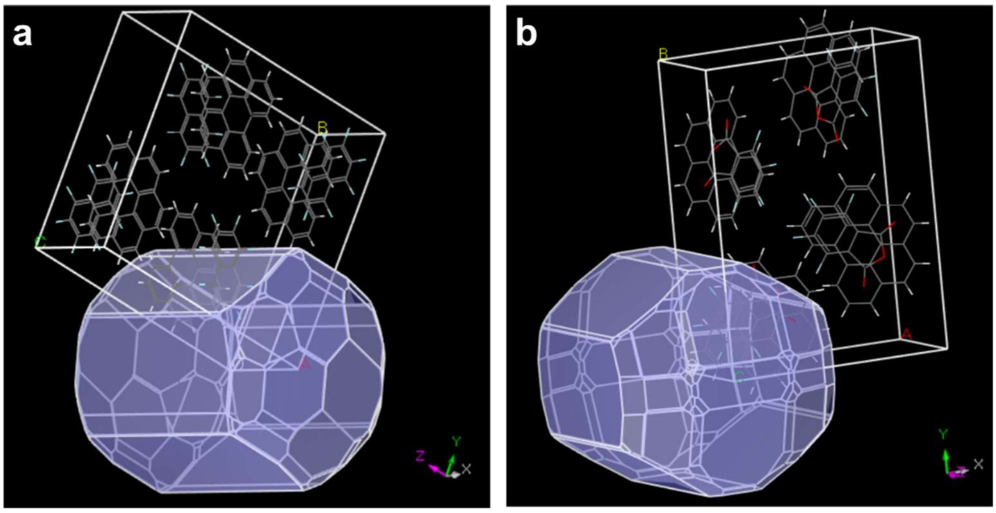

Supplementary Figure 6. Predicted equilibrium morphology of cocrystals. (a) The simulated equilibrium morphology of BGP-OFN co-crystal, (b) The simulated equilibrium morphology of BGP-TFPA co-crystal, based on the attachment energies.

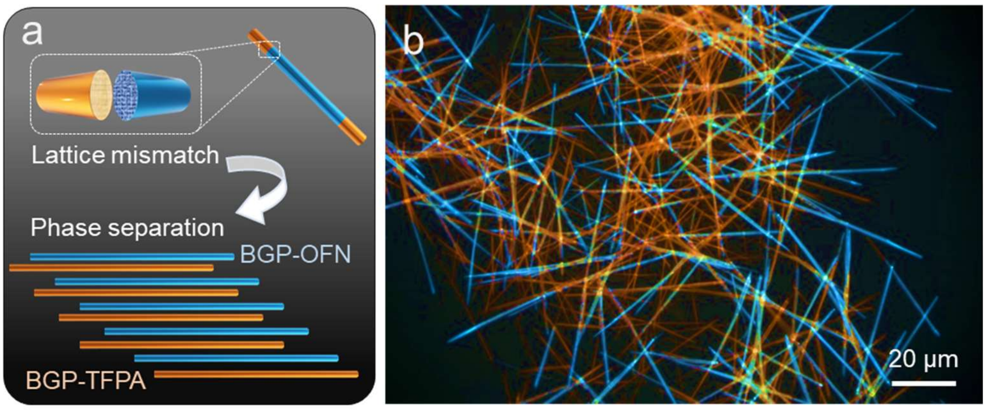

Supplementary Figure 7. Phase separation of multicomponent. (a) Schematic diagram of the lattice mismatch and phase separation during the co-assembly process of BGP-OFN and BGP-TFPA cocrystals (b) Corresponding FM images of BGP-OFN and BGP-TFPA cocrystal.

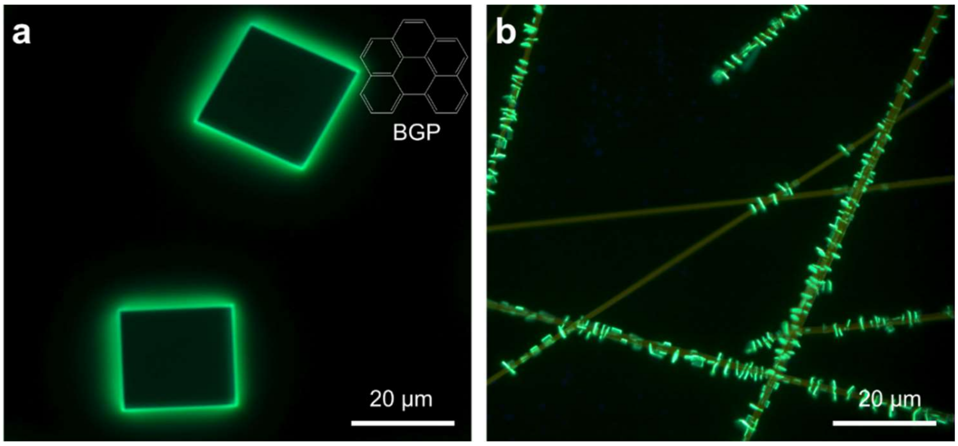

Supplementary Figure 8. Morphology of the organic microcrystals. (a) FM images of BGP crystal micro sheets, (b) FM images of binary heterostructures of BGP sheets and BGP-$OFN_{(1-x)}$-$TFPA_{(X)}$ alloy microwires prepared at x = 25% when excited by UV light.

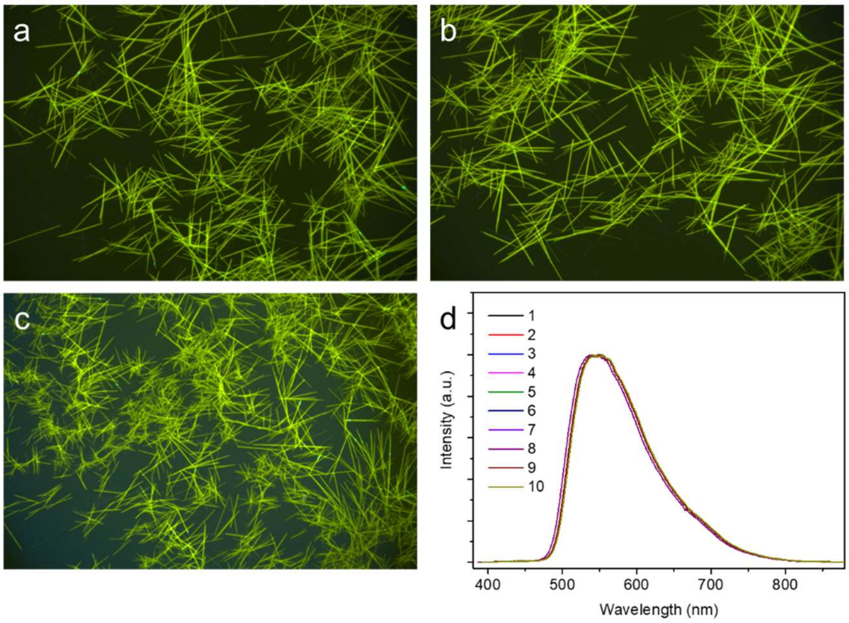

Supplementary Figure 9. Morphology and optical characterizations of alloys. (a-c) FM images of BGP-$OFN_{(0.8)}$-$TFPA_{(0.2)}$ organic alloy nanowires. (d) The corresponding spatially resolved PL spectra of obtained BGP-$OFN_{(0.8)}$-$TFPA_{(0.2)}$ nanowires on different area of substrate.

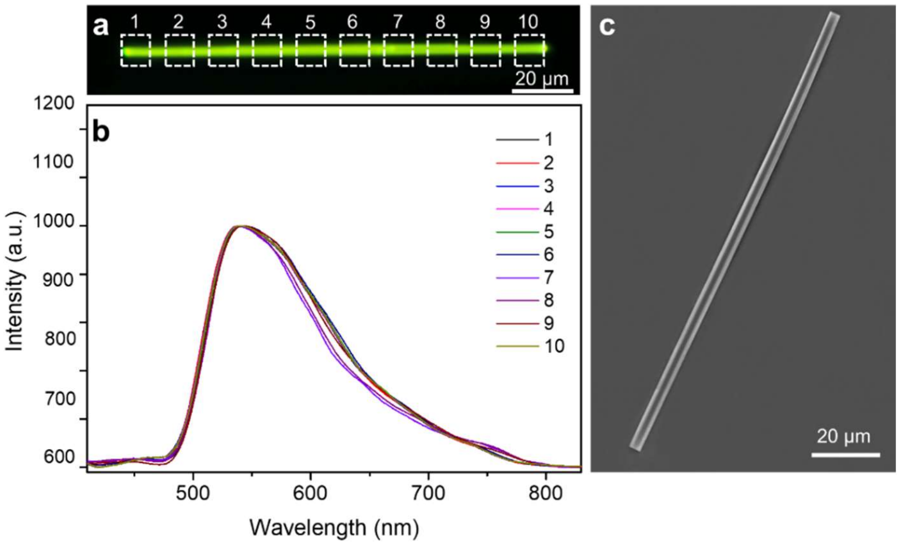

Supplementary Figure 10. Optical characterizations of organic alloy. (a) FM images of BGP-OFN$_{(0.8)}$-TFPA$_{(0.2)}$ alloy microwire. The scale bar is 20 μm. (b) PL spectra collected from different sections marked in FM images of BGP-OFN$_{(0.8)}$-TFPA$_{(0.2)}$ alloy excited by laser beam λ = 375 nm. (c) SEM images of BGP-OFN$_{(0.8)}$-TFPA$_{(0.2)}$ alloy microwire. The scale bar is 20 μm.

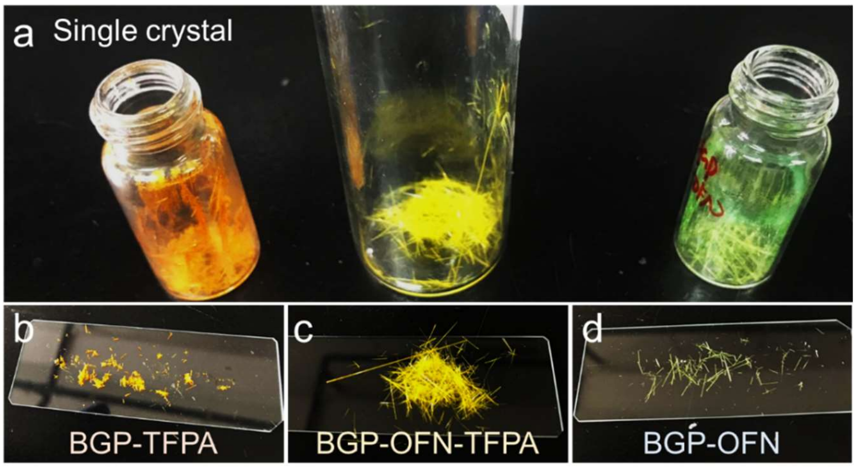

Supplementary Figure 11. Single crystals of cocrystals and alloy. (a) The single crystal obtained by slowly evaporation. (b) Photo of BGP-TFPA single crystal with orange color. (c) Photo of BGP-OFN$_{(0.8)}$-TFPA$_{(0.2)}$ single crystal with olive color. (d) Photo of BGP-OFN single crystal with blue-green color.

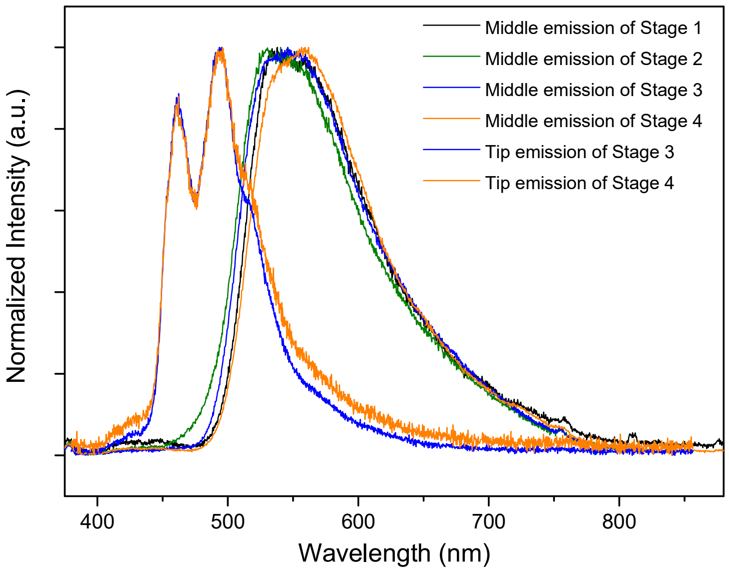

Supplementary Figure 12. Optical characterization of triblock nanowires. Four growth stages: the emission spectra of blue-olive-blue triblock heterostructure at each stage.

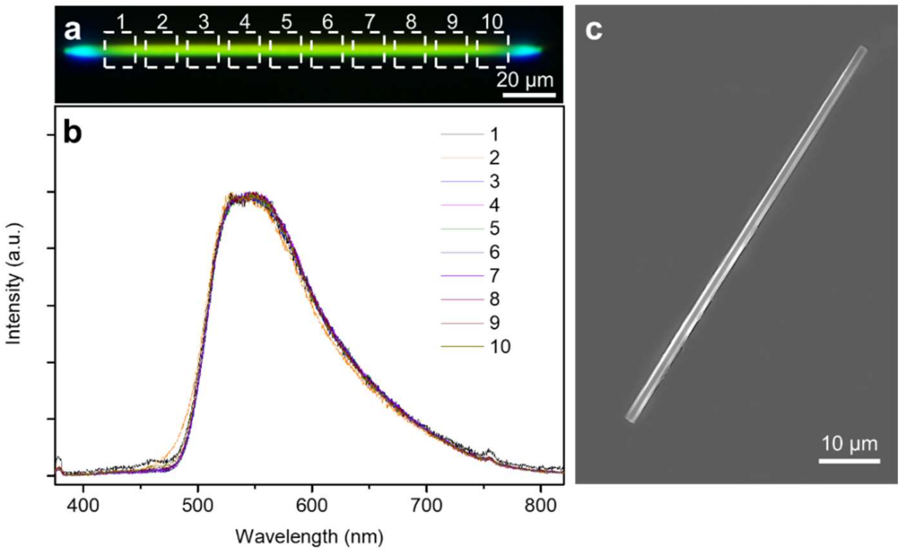

Supplementary Figure 13. Optical characterization of triblock nanowires. (a) FM images of blue-olive-blue triblock. The scale bar is 20 μm. (b) PL spectra collected from different sections marked in FM images of central part in triblock heterostructure microwire excited by laser beam λ = 375 nm. (c) SEM images of blue-olive-blue triblock. The scale bar is 10 μm.

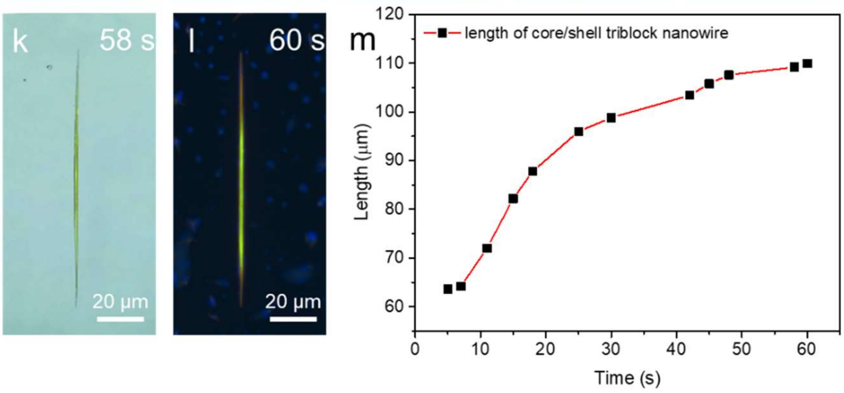

Supplementary Figure 14. Growth process of core/shell triblock nanowires. (a-k) The self-assembled growth process of a typical orange-olive/orange-organic triblock nanowire recorded by a bright-field microscope with a scale bar of 20 μm. (l) The fluorescence microscopy image of corresponding as-prepared orange-olive/orange-organic triblock nanowires. (m) The time-dependent length of the whole core/shell triblock nanowire.

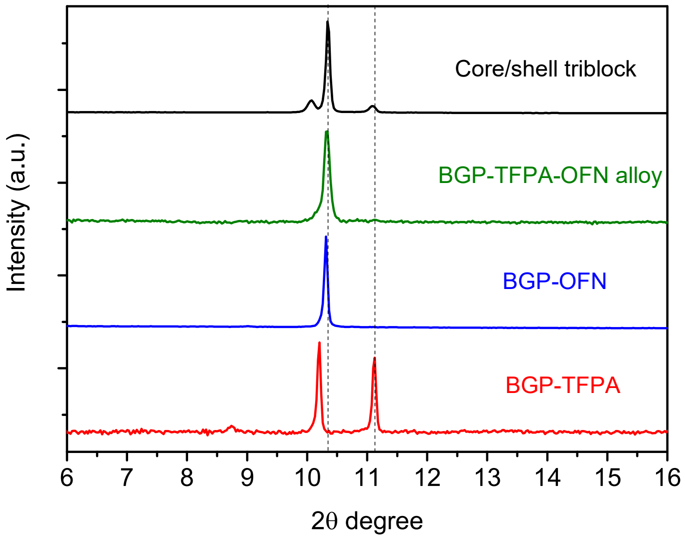

Supplementary Figure 15. Structure character of the organic nanowires. XRD patterns of BGP-TFPA cocrystal, BGP-OFN cocrystal, BGP-OFN$_{(0.8)}$-TFPA$_{(0.2)}$ alloy and Core/shell triblock heterostructure.

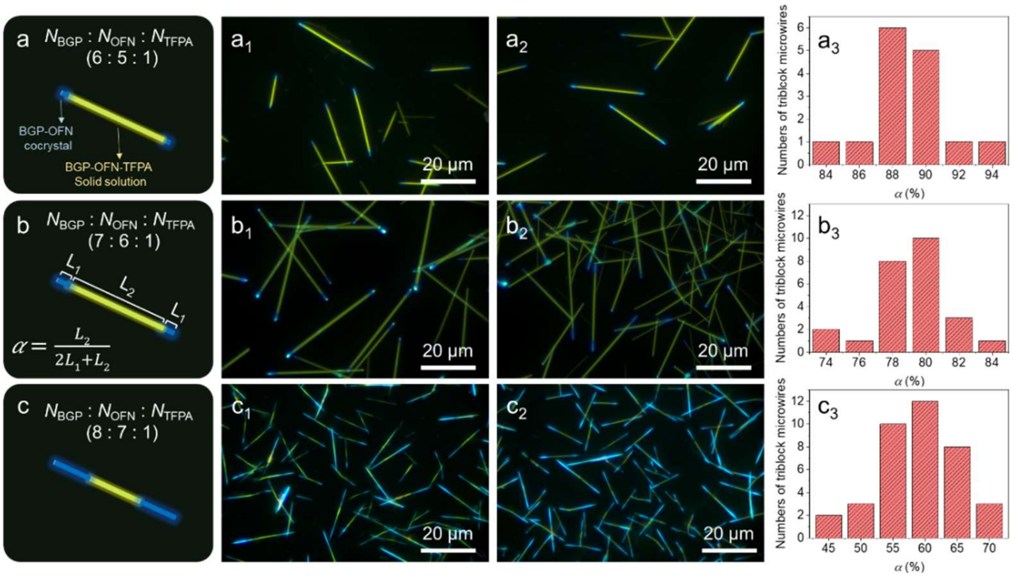

Supplementary Figure 16. Controlled synthesis of triblock nanowires. (a-c) Schematic diagram of the blue-olive-blue triblock heterostructures in the different molar ratios, and the length ratio of blue and olive emission segments. FM images of triblock microwires with the α of 88-90% (a_1, a_2) (adjacent areas of quartz substrate), 78-80% (b_1, b_2) and 55-65% (c_1, c_2) in the different molar ratios. (a_3, b_3, c_3) A statistic histogram of the α distribution of the as-prepared triblock microwires, respectively.

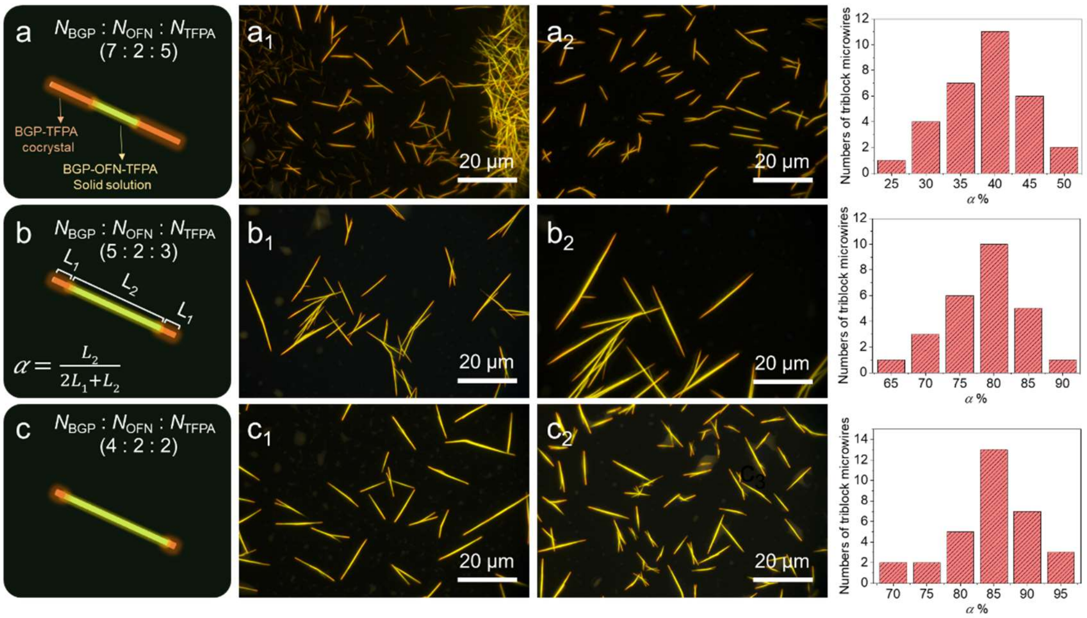

Supplementary Figure 17. Controlled synthesis of core/shell triblock nanowires. (a-c) Schematic diagram of the orange-olive/orange-orange triblock heterostructures in the different molar ratios, and the length ratio of core and shell segments. FM images of triblock microwires with the α of 35-45% (a_1, a_2) (adjacent areas of quartz substrate), 75-85% (b_1, b_2) and 85-90% (c_1, c_2) in the different molar ratios. (a_3, b_3, c_3) A statistic histogram of the α distribution of the as-prepared triblock microwires, respectively.

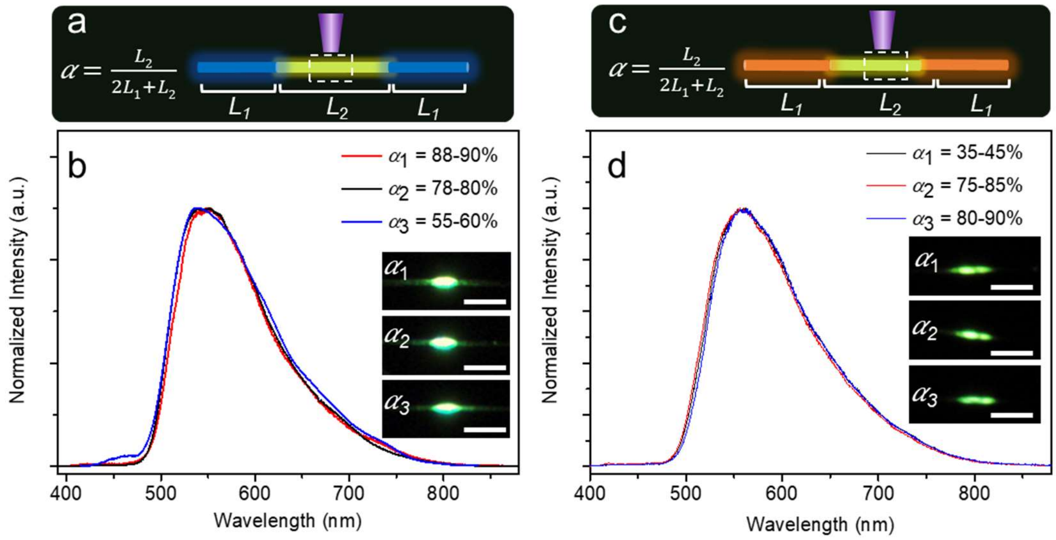

Supplementary Figure 18. Optical characterizations of triblock nanowires. (a, c) Schematic diagram of the triblock heterostructures, and the length ratio of two segments. (b) Spatially resolved PL spectra of blue-olive-blue triblock with different length ratio, which recorded at the middle sections marked in (a). Insets are the corresponding FM images excited by laser beam λ = 375 nm. (d) Spatially resolved PL spectra of orange-olive/orange-orange triblock with different length ratio, which recorded at the middle sections marked in (c). Insets are the corresponding FM images excited by laser beam λ = 375 nm.

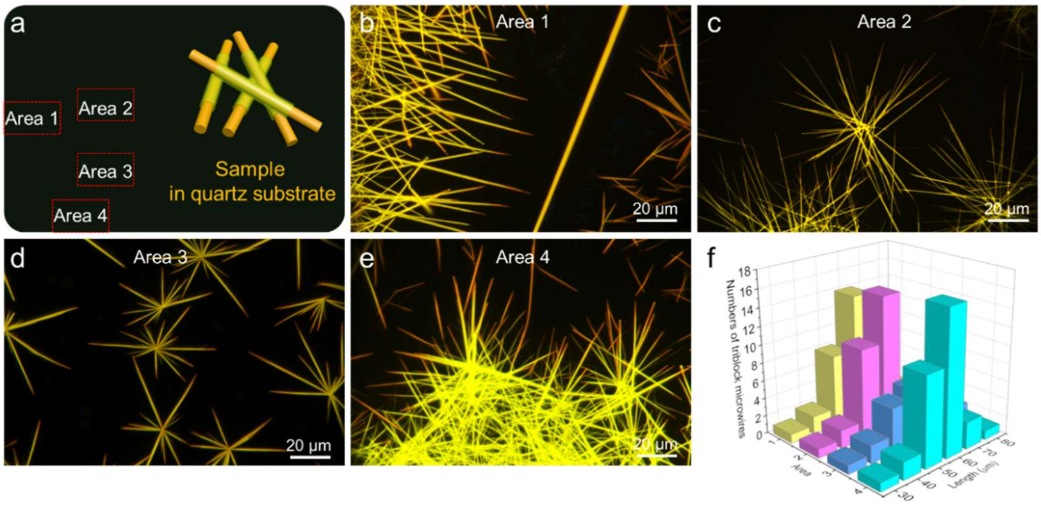

Supplementary Figure 19. Large-scale synthesis of core/shell triblock nanowires. (a) Schematic diagram of the distributions of orange-olive/orange-orange core/shell triblock heterostructures in different areas of the quartz substrate. Fluorescence micrographs of triblock in the area 1 (b), area 2 (c), area 3 (d) and area 4 (e). (f) The histogram of length distribution of triblock heterostructures, by randomly measuring the FM images of a large number of microwire samples. The length of the triblock microwires ranges from 50 to 60 μm.

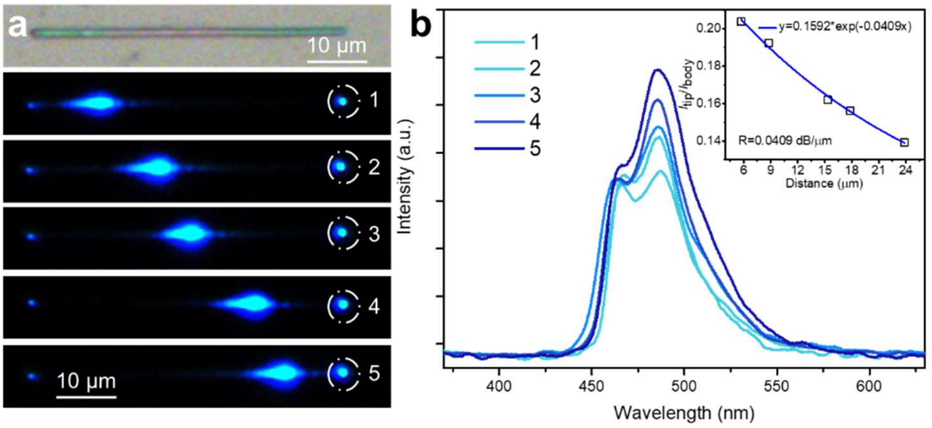

Supplementary Figure 20. Optical waveguide properties of the BGP-OFN cocrystals. (a) Bright-field image of BGP-OFN, and PL images by exciting the BGP-OFN microwire at different points with 375 nm laser. The scale bar is 10 μm. (b) Spatially resolved PL spectra from the tip of the microwire for different separation distances between the exciting point and the right tip of the microwire shown in (a).

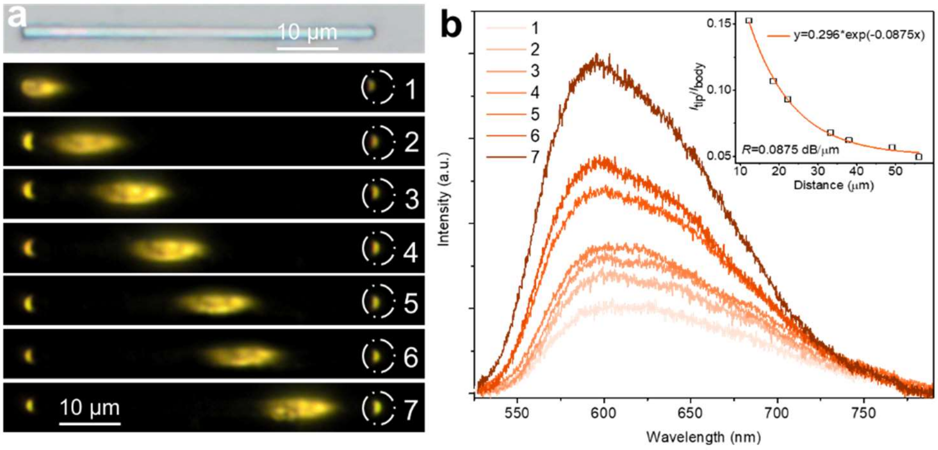

Supplementary Figure 21. Optical waveguide properties of the BGP-TFPA cocrystals. (a) Bright-field image of BGP-TFPA, and PL images by exciting the BGP-TFPA microwire at different points with 375 nm laser. The scale bar is 10 μm. (b) Spatially resolved PL spectra from the tip of the microwire for different separation distances between the exciting point and the right tip of the microwire shown in (a).

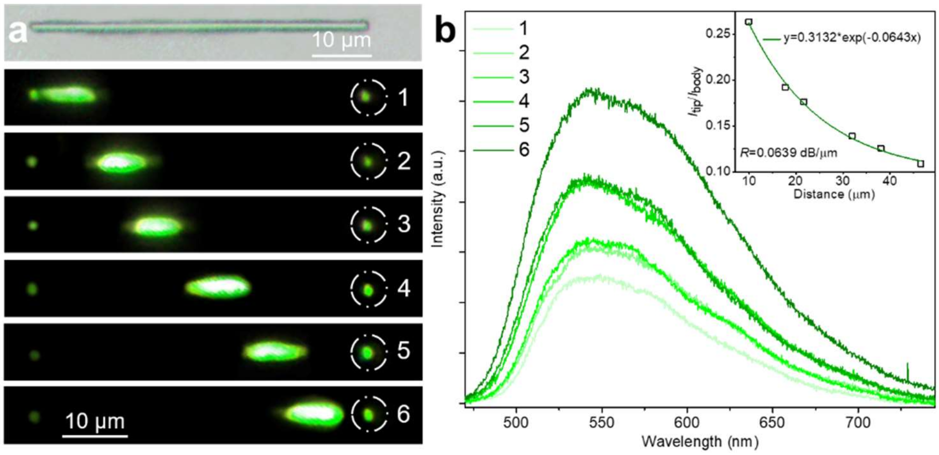

Supplementary Figure 22. Optical waveguide properties of the organic alloys. (a) Bright-field image of BGP-OFN$_{(0.8)}$-TFPA$_{(0.2)}$ alloy, and PL images by exciting the BGP-OFN$_{(0.8)}$-TFPA$_{(0.2)}$ alloy microwire at different points with 375 nm laser. The scale bar is 10 μm. (b) Spatially resolved PL spectra from the tip of the microwire for different separation distances between the exciting point and the right tip of the microwire shown in (a).

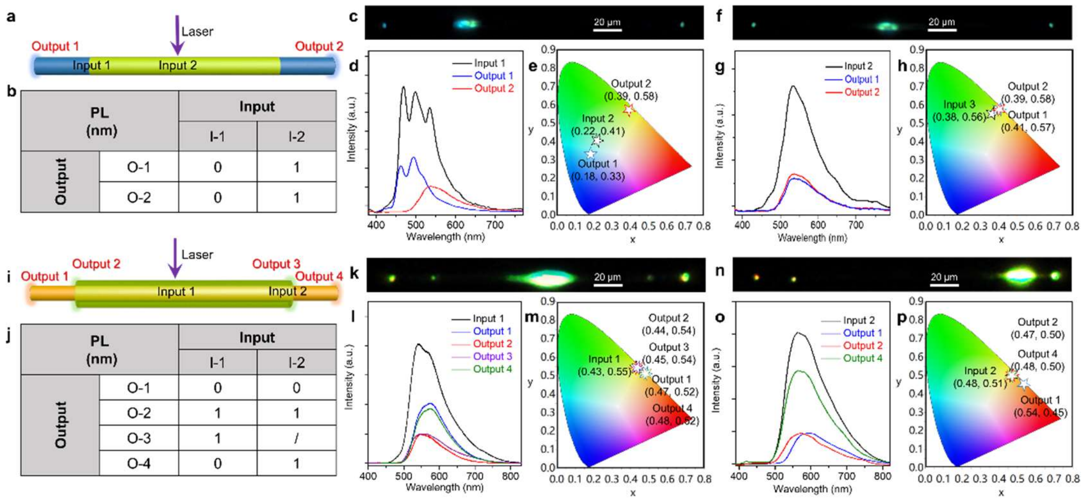

Supplementary Figure 23. **Optical applications of triblock nanowires.** Photonic signal logic operation based on two types of triblock nanowire. (a) Schematic diagram of the blue-olive-blue triblock under a microarea excitation at two different positions with a 375 nm laser. (b) The corresponds to truth table for the optical logic operations information code. (c, f) FM images of triblock nanowires under Input 1 and Input 2 excitation as indicated in (a), respectively. (d, g) The collected PL spectrum of different positions when the nanowires excited at different positions as indicated in (a). (e, h) Corresponding CIE chromaticity diagram of emission color. (i) The illustration for the optical logic gate based on the orange-orange/olive-orange THSs. (j) The truth table for the optical logic gate shown in (i). (k, n) FM images of triblock nanowires excited at different ports. (l, o) The collected PL spectrum of different output and input ports when the different input position in (i) were excited. (m, p) Corresponding CIE chromaticity diagram.

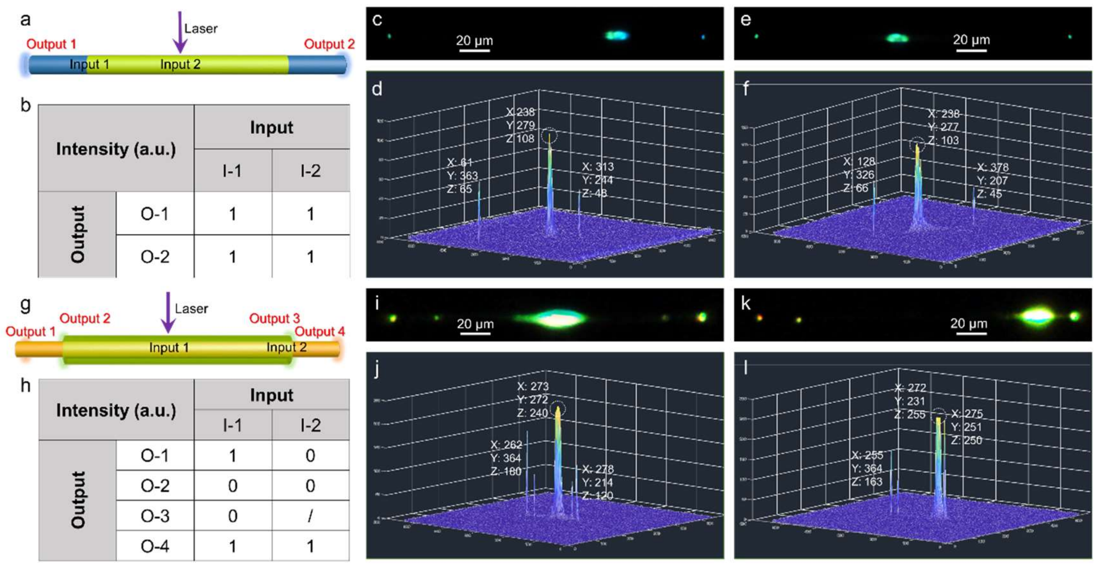

Supplementary Figure 24. Optical applications of triblock nanowires. (a) Schematic diagram of the individual triblock nanowire under excitation at two different positions with a 375 nm laser. (b) The logic table display intensity signal output's codes. (c, e) FM images at inputs I-1 and I-2. (d, f) The light intensity distribution of FM images obtained by Matlab simulation. (g) Schematic diagram of the individual core/shell triblock under excitation at two different positions with a 375 nm laser. (h) The logic table display intensity signal output's codes. (i, k) FM images at inputs I-1 and I-2 of core/shell nanowire. (j, l) The light intensity distribution of FM images (i, k) obtained by Matlab simulation.

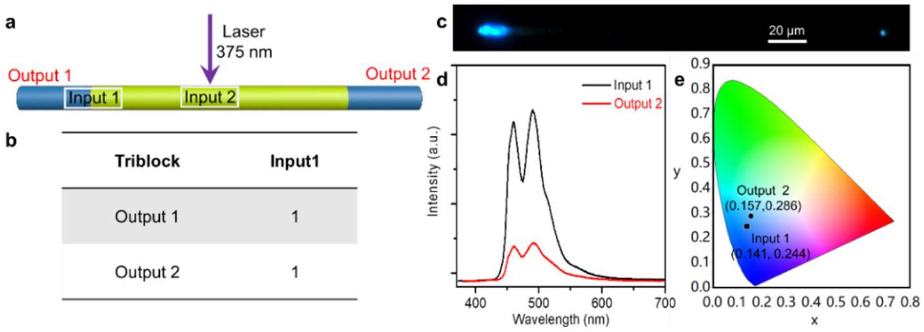

Supplementary Figure 25. Optical applications of triblock nanowires. The photonic signal processing of triblock heterostructure. (a) Schematic diagram of the blue-olive-blue triblock under a microarea excitation at Input1 positions with a 375 nm laser. (b) The corresponds to truth table for the optical logic operations information code. (c) FM images of triblock microwires under Input 1 excitation as indicated in (a). The scale bar is 20 μm. (d) The PL spectra collected at the tips of the microwires excited at Input1 positions as indicated in (a). (e) Corresponding CIE chromaticity diagram of emission color.

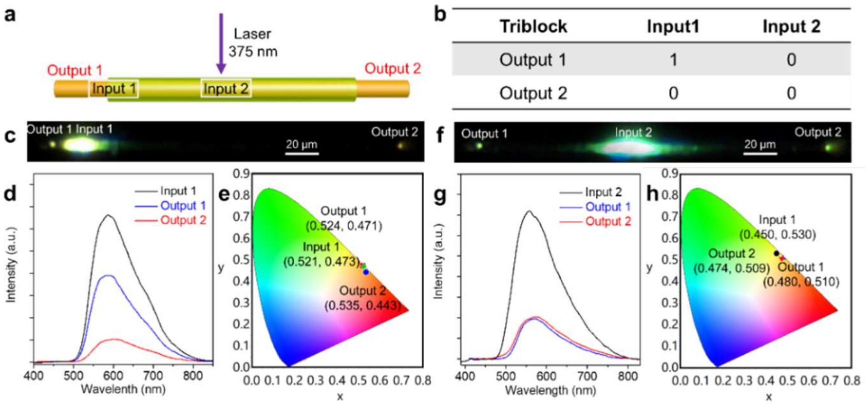

Supplementary Figure 26. Optical applications of core/shell triblock nanowires. The photonic signal processing of core/shell triblock heterostructure. (a) Schematic diagram of the core/shell triblock under a microarea excitation with a 375 nm laser. (b) The corresponds to truth table for the optical logic operations information code. (c, f) FM images of triblock microwires under Input 1 and Input 2 excitation as indicated in (a), respectively. The scale bar is 20 μm. (d, g) The PL spectra collected at the tips of the microwires excited at different positions as indicated in (a). (e, h) Corresponding CIE chromaticity diagram of emission color.

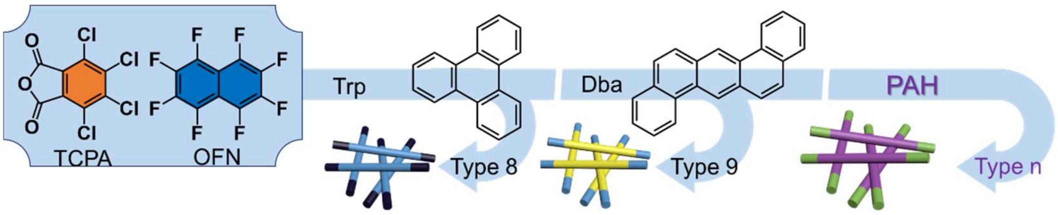

Supplementary Figure 27. Molecular structures of organic heterostructure nanowires.

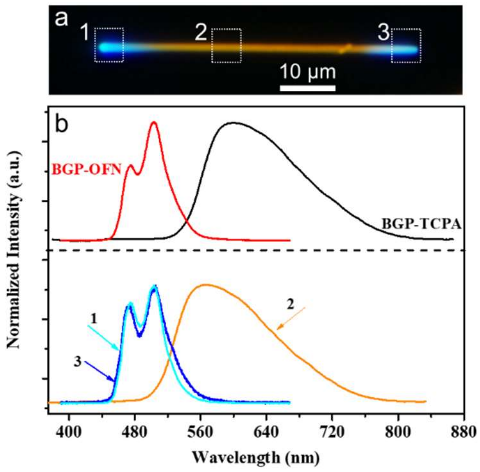

Supplementary Figure 28. Optical characterizations of triblock nanowires. (a) FM image of individual blue-orange-blue triblock nanowire. The scale bar is 10 μm. (b) PL spectra collected from different sections marked in (a).

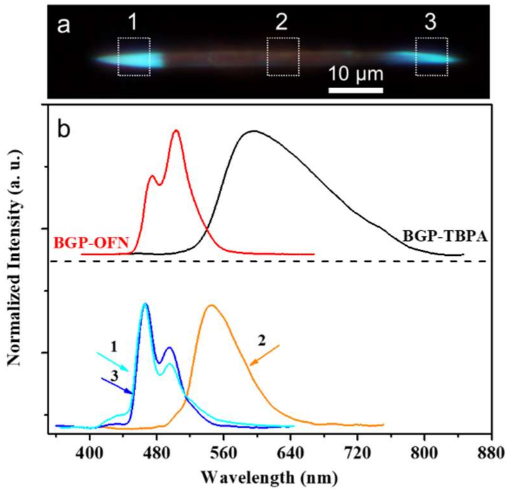

Supplementary Figure 29. Optical characterizations of triblock nanowires. (a) FM image of individual blue-brown-blue triblock nanowire. The scale bar is 10 μm. (b) PL spectra collected from different sections marked in (a).

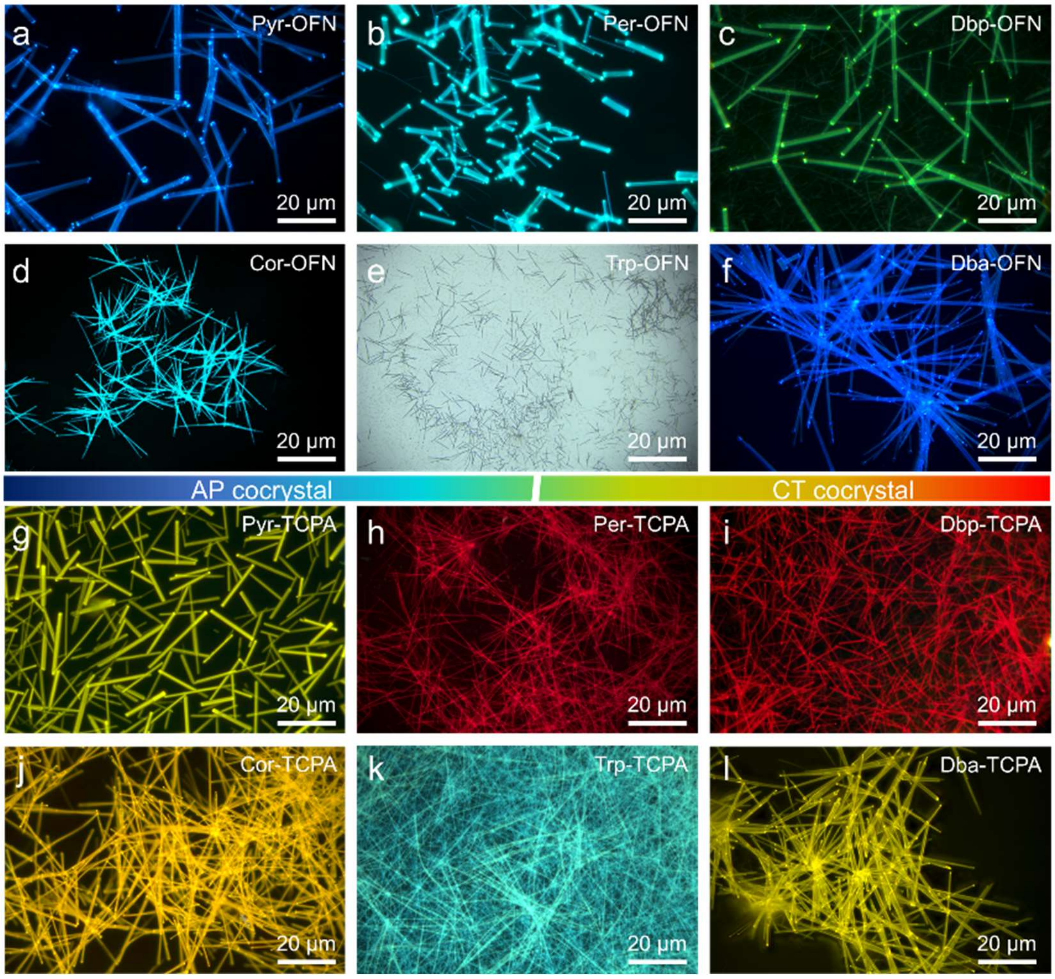

Supplementary Figure 30. Morphologies of organic cocrystals. (a-f) FM images of various AP cocrystal nanowires based on the polycyclic aromatic hydrocarbons (PAH) and OFN molecules. (g-l) FM images of various CT cocrystal nanowires based on the PAH and TCPA molecules. All scale bar are 20 μm.

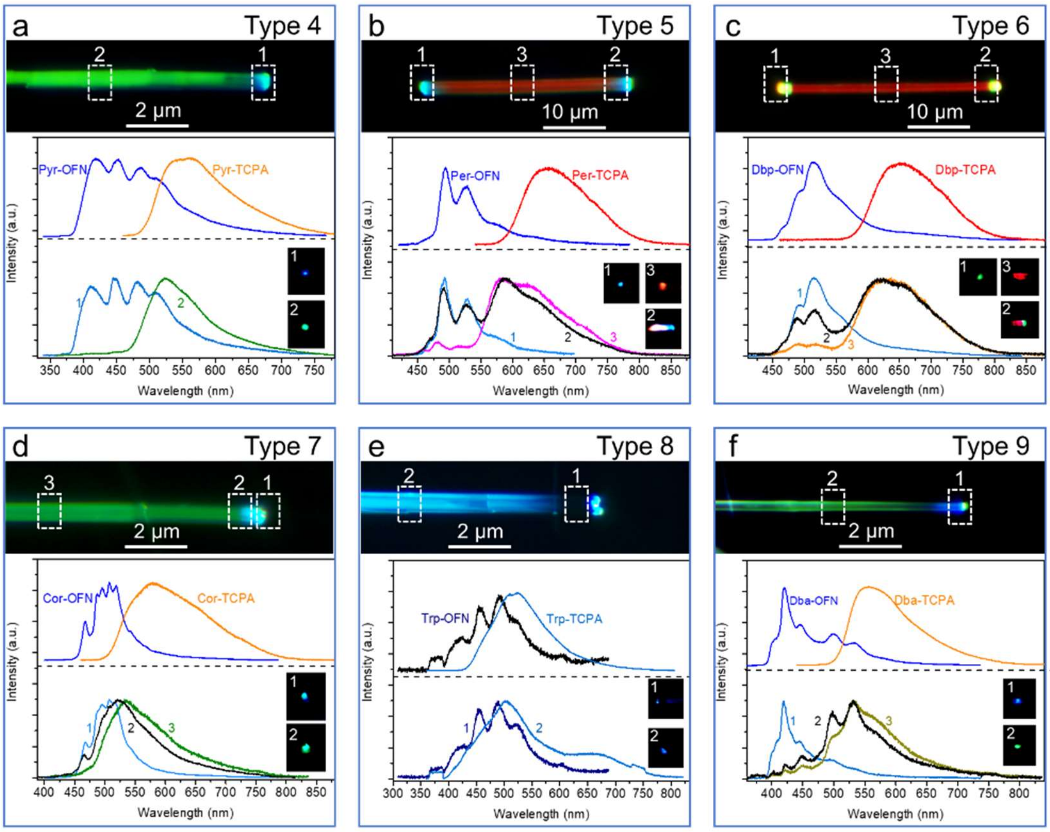

Supplementary Figure 31. Optical characterizations of triblock nanowires. (a-f) Optical characterization of various heterostructures nanowires. (top image) FM image of individual triblock nanowire. The scale bar is 2 μm. (bottom image) PL spectra collected from different sections marked in (top image).

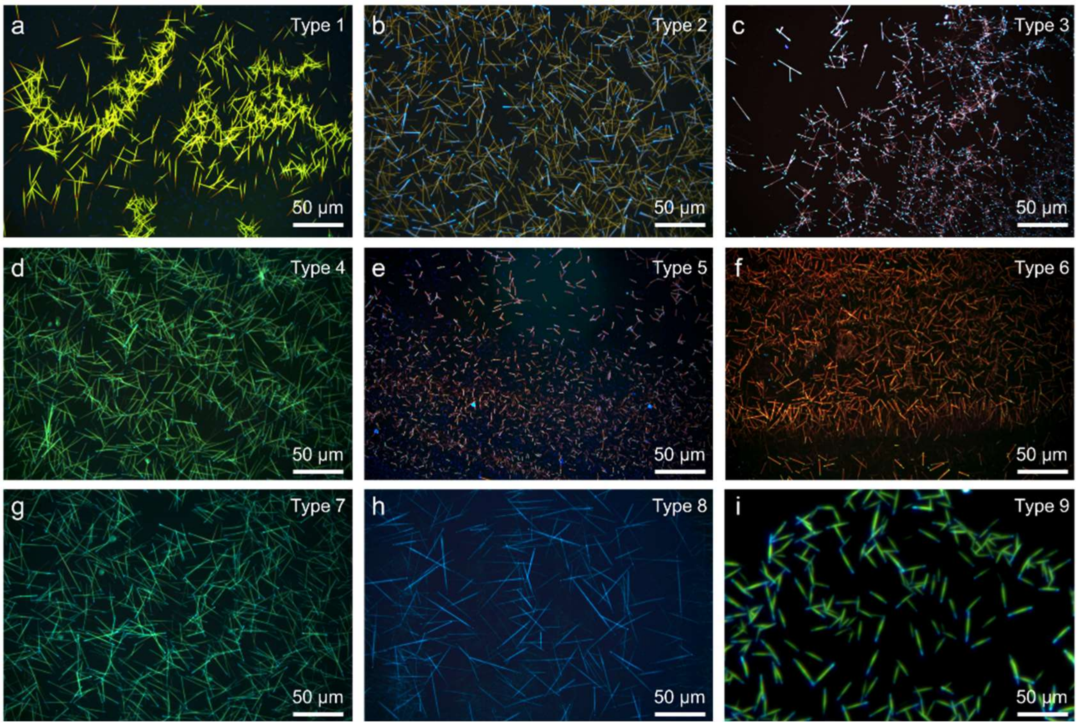

Supplementary Figure 32. Morphologies of the triblock nanowires. FM images of the large-scale synthesis of triblock nanowires.

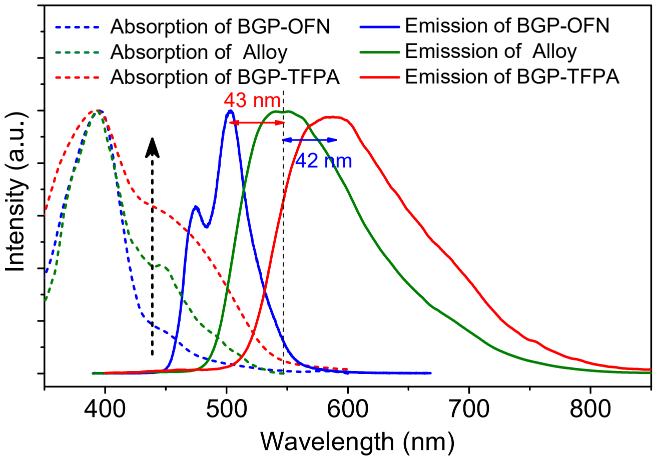

Supplementary Figure 33. Optical characterizations of organic crystals. The diffuse absorption and PL spectra of BGP-OFN, BGP-TFPA and BGP-OFN$_{(0.8)}$-TFPA$_{(0.2)}$ alloy.

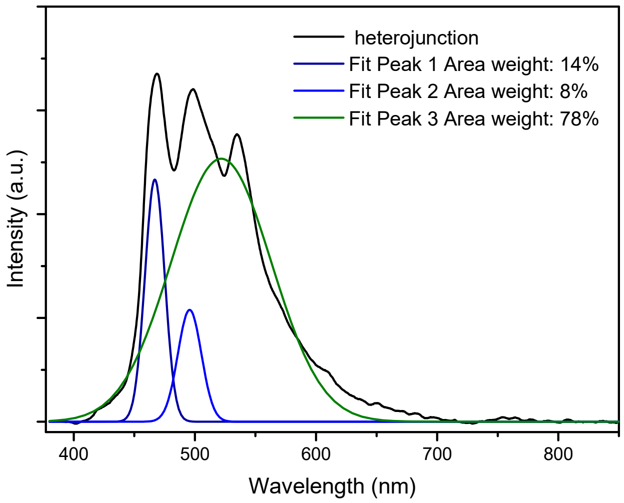

Supplementary Figure 34. Optical characterizations of organic crystals. The PL spectra collected at the heterojunction of the microwires by fitting the spectra with gaussian bands.

Supplementary Table 1. Crystal data and structure refinement for BGP-OFN and BGP-TFPA.

Name	BGP-OFN	BGP-TFPA
CCDC Number	1964760	2058999
Empirical formula	$C_{32}H_{12}F_8$	$C_{30}H_{12}F_4O_3$
Formula weight	548.44	496.40
Temperature	193.0 K	193.0 K
Wavelength	0.71073 Å	1.34139 Å
Crystal system	Monoclinic	Orthorhombic
Space group	P 2_1/n	P na2_1
a (Å)	6.7334(6)	14.7390(2)
b (Å)	18.2843(18)	19.6720(3)
c (Å)	18.2556(18)	7.13870(10)
a (°)	90	90
b (°)	98.535(3)	90
g (°)	90	90
Cell Volume ($Å^3$)	2222.7(4)	2069.83(5)
Z: 4 Z': 0	Z: 4 Z': 0	Z: 4 Z': 0
Density (g/cm^3)	1.639	1.593
R-Factor (%)	8.07	4.97

Supplementary Table 2. Attachment energies of various crystal facets (hkl) of BGP-OFN cocrystal calculated by using the Material Studio Package.

(hkl)	d_{hkl} (Å)	E_{att} (total)/ (kcal mol^{-1})	% Total facet area
(011)	12.847	-50.430	74.466
(020)	9.142	-63.717	6.620
(10-1)	6.572	-117.078	13.600
(110)	6.625	-129.417	3.385
(11-1)	6.185	-128.040	0.558
(101)	5.966	-133.923	1.369
(120)	5.382	-146.824	---

Supplementary Table 3. Attachment energies of various crystal facets (hkl) of BGP-TFPA cocrystal calculated by using the Material Studio Package.

(hkl)	d_{hkl} (Å)	E_{att} (total)/ (kcal mol^{-1})	% Total facet area
(110)	11.796	-57.924	59.484
(020)	9.836	-63.195	16.875
(011)	6.711	-109.502	11.556
(01-1)	6.711	-109.502	11.556
(111)	6.107	-125.630	0.264
(11-1)	6.107	-125.630	0.264
(12-1)	5.379	-138.183	---

Supplementary Table 4. Surface energies of various crystal facets (hkl) of BGP-OFN calculated by using the Material Studio Package.

(hkl)	d_{hkl} (Å)	E_{surf} (total)/ (kcal mol^{-1})	% Total facet area
(011)	12.847	0.146	7.155
(020)	9.142	0.131	21.417
(002)	9.027	0.149	0.642
(021)	8.156	0.150	0.372
(012)	8.094	0.134	33.969
(10-1)	6.572	0.175	6.011
(110)	6.257	0.186	1.008
(11-1)	6.184	0.182	6.840
(101)	5.967	0.184	2.018
(111)	5.672	0.188	3.179
(11-2)	5.515	0.192	0.502
(120)	5.382	0.191	2.168
(12-1)	5.336	0.191	1.075

Supplementary Table 5. Surface energies of various crystal facets (hkl) of BGP-TFPA calculated by using the Material Studio Package.

(hkl)	d_{hkl} (Å)	*E*surf (total)/ (kcal mol^{-1})	% Total facet area
(110)	11.796	0.165	1.184
(020)	9.836	0.150	9.242
(120)	8.181	0.146	32.396
(200)	7.370	0.154	16.278
(210)	6.901	0.168	0.268
(011)	6.711	0.180	6.262
(111)	6.107	0.191	4.601
(121)	5.379	0.197	0.364
(20-1)	5.127	0.200	0.797
(21-1)	4.962	0.199	1.444
(131)	4.589	0.196	1.089

Supplementary Note 1: Optical logic gate device prototype based on two types of triblock organic heterostructures.

In order to understand the photon propagation of single-component nanowires, the micro-area photoluminescence microscopy images and distance-dependent emission spectra are performed to investigate the optical waveguide nature of the BGP-OFN (Supplementary Figure 20), BGP-TFPA (Supplementary Figure 21) and alloy nanowires (Supplementary Figure 22). The optical loss coefficients (α) are estimated based on the $I_{tip}/I_{body} = A \exp(-\alpha D)$[1], Where D is the propagation distance of photon, the resulted α values are 0.0409 dB μm^{-1}, 0.0875 dB μm^{-1} and 0.0639 dB μm^{-1} corresponding to BGP-OFN, BGP-TFPA cocrystal and alloy. Based on the advantages of lower optical loss of these nanowires, the THSs nanowires with the excitation position-dependent multicolor emission characteristics and multiple input/output channels prompt us to perform optical signal processing at microstructure, such as optical logic gate[2,3]. As shown in Supplementary Figure 23a, the blue-olive-blue THSs consist of two optical channels (Out1 and Out2) locating on the tip, two inputs (Input1 and Input2) at the junctions and center positions, respectively, where

the excitation by the same UV laser beam but at different input sections of the nanowire resulting in the optical signals with a specific wavelength at output. When excited at Input1 (Supplementary Figure 23c), owing to the photon propagation from junction to the two tips are passive waveguide, thus the out-coupled emitting signal Output1 at 470 and 500 nm corresponding to the emission of the BGP-OFN, and the Output2 at 550 nm corresponding to the emission of alloy (Supplementary Figure 23d). The corresponding CIE coordinates from the emission spectrum were further calculated to well define the characteristics of the emitted light at each position in the THSs nanowire (Supplementary Figure 23e). When the excitation position moved to the central part (Input2), the emission color at the two tips is consistent with the excited position (Supplementary Figure 23f-h). In contrast with the triblock nanowires, the core/shell THSs have four output channels located on two tips and two junctions as shown in the Supplementary Figure 23i. When excited at middle part (Input 1) in nanowire, the Output 1 and Output 4 are bright, and the Output 2 and Output 3 are dim (Supplementary Figure 23k). We speculate that is because the most of the photons are coupled out through the core and only a small part of the photons is emitted at the junction. At the same time, due to the passive propagation of photons, the spectrum of the heterojunction corresponds to the shell emission and the spectrum of the port corresponds to the core emission (Supplementary Figure 23i). When excited at Input 2, except the Output 3, other output terminals are bright (Supplementary Figure 23e), and the collected spectrum at each position is shown in Supplementary Figure 23o. Moreover, the aggregation induced red shift, the PL spectrum at output 1 is significantly different from the excitation position. Furthermore, the spectral is further defined into CIE coordinates (Supplementary Figure 23p). So that, the excitation position-dependent multicolor emission properties with the selective wavelength of the blue-olive-blue triblock nanowires and multi-channel output emission of the orange-olive/orange-olive core/shell triblock nanowires were applied in the optical logic gate operation at nanoscale. In the blue-olive-blue triblock model, both of output 1 and 2 go through strong light signal. If the

output position and the input position have the same emission spectrum, the optical signal is defined as "1", otherwise the signal is defined as "0", then an optical logic circuit table can be obtained in Supplementary Figure 23b. As for the orange-olive/orange-olive core/shell triblock nanowire, the logic gate of individual nanowire follows the similar coding procedure, except that the optical signal has four output channels, in which the signal operation is summarized in Supplementary Figure 23j. Another two optical logic gate modes also summarized in Supplementary Figures 25 and 26. By this way, an optical logic gate could be achieved based on triblock nanowire with multicolor/channel, which provides a potential application for the nanophotonic circuits.

We further use Matlab software to calculate the light intensity distribution of each pixel of the FM image, and extract it to generate a three-dimensional image, where the z-axis value is based on the light intensity (Supplementary Figure 24). It can be seen that the z-axis values of input position keep the highest, while the z-axis value of the tips of nanowire is the middle due to the optical loss and self-absorption, which is coded as "signal 1", the low optical signal is defined "0". So that, we readily construct the optical logic gate with multiple input/output channels by Matlab calculation based on the light intensity of FM images of individual heterostructures nanowire under excitation at different position with a 375 nm laser.

Supplementary Note 2: Quantitative analysis of the optical data.

Take the absorption and PL spectrum of cocrystals, alloys and heterojunction as an example, we further perform a more quantitative analysis of the optical data, for example by fitting the spectra with gaussian bands and report spectral shifts or spectral weights in a more quantitative and consistent way (Supplementary Figures 33 and 34).

Supplementary References:

1. Zhuo, M. P.; Tao, Y. C.; Wang, X. D.; Wu, Y.; Chen, S.; Liao, L. S.; Jiang, L. 2D organic photonics: an asymmetric optical waveguide in self-assembled halogen-bonded cocrystals. *Angew. Chem. Int. Ed.* **2018**, *130*, 11470–11474.
2. Yao, W.; Yan, Y.; Xue, L.; Zhang, C.; Li, G.; Zheng, Q.; Zhao, Y. S.; Jiang, H.; Yao, J. Controlling the structures and photonic properties of organic nanomaterials by molecular design. *Angew. Chem. Int. Ed*. **2013**, *52*, 8713–8717.
3. Takazawa, K. Micrometer-Sized Rings Self-assembled from Thiacyanine Dye Molecules and Their Waveguiding Properties. *Chem. Mater.* **2007**, *19*, 5293–5301.
4. Zhuo, M. P.; Wu, J. J.; Wang, X. D.; Tao, Y. C.; Yuan, Y.; Liao, L. S. Hierarchical self-assembly of organic heterostructure nanowires. *Nat. Commun*. **2019**, *10*, 3839.
